# Effect of Electrodeposition Parameters on the Morphology, Topography and Corrosion Resistance of Epoxy Resin/Zinc Hybrid Coatings

**DOI:** 10.3390/ma14081991

**Published:** 2021-04-15

**Authors:** Alina Crina Mureşan, Daniela Laura Buruiană, Gina Genoveva Istrate, Ştefan Cătălin Pintilie

**Affiliations:** 1 Interdisciplinary Research Center in the Field of Eco-Nano Technology and Advanced Materials (CC-ITI), Faculty of Engineering, Dunarea de Jos University of Galati, 47 Domneasca Street, 800008 Galati, Romania; daniela.buruiana@ugal.ro (D.L.B.); gina.istrate@ugal.ro (G.G.I.); 2 Cross-Border Faculty, Department of Applied Sciences, Dunarea de Jos University of Galati, 47 Domneasca Street, 800008 Galati, Romania; Stefan.pintilie@ugal.ro

**Keywords:** epoxy resin/zinc coatings, electrodeposition, topography, corrosion resistance, sulphate reducing bacteria

## Abstract

The paper presents the morphology, topography and corrosion behavior of epoxy resin/zinc hybrid coatings obtained by electrodeposition from zinc sulphate electrolyte. The effect of current density and mean diameter size of polymer particles used for electrodeposition of the epoxy resin/zinc coatings on the morphology, topography and roughness of the surfaces were investigated by Scanning Electron Microscopy and Atomic Force Microscopy. The corrosion behavior of the hybrid coatings in 0.5 M sodium chloride solution was evaluated using electrochemical methods. For microbiological corrosion, the effect of sulphate reducing bacteria (SRB) on the surfaces was evaluated using epifluorescence microscopy. The surface roughness before and after bacteria attachment was evaluated using Atomic force microscopy. Polymer particles provide an influence in restricting the growth of zinc crystals as well as a catalytic role in nucleation sites increase. The results of electrochemical tests indicate a very good corrosion resistance of hybrid coatings comparing with zinc coatings. Epifluorescence images demonstrate that hybrid coating surfaces are slightly attacked by sulphate reducing bacteria.

## 1. Introduction

Electrodeposition is an important industrial process used for protection of steel from corrosion. Zinc coatings are often used for protection of steel and iron from corrosion, due to the “sacrificial” role of zinc, low cost, easy application and good corrosion properties in natural atmosphere. Under an aggressive corrosive environment (industrial, marine, rural and urban atmosphere environments), the corrosion properties of zinc coatings dramatically decrease [1,2,3,4]. For this reason, it is necessary to improve these coatings by adding different organic and inorganic particles. For example, it was reported that new composite coatings were obtained by co-deposition using metal matrix and different particles such as SiO_2_, TiO_2_, ZrO_2_ nanoparticles, carbon nanotubes, polytetrafluorethylene (PTFE), polyaniline (PAN), polymethylmethacrylate (PMMA) exhibited improved corrosion properties when compared with conventional zinc [5,6,7,8,9,10]. In these composite coatings, mixed layers of corrosion products from the metal and particles in the composite coating often appear and demonstrate a synergistic interaction which contributes to the improvements in properties of hybrid layers.

Polymers like epoxy resin are usually used as polymeric coatings because they separate the steels from external environments and serve as a barrier to protect steel surface areas from assertive environmental attack. Their good protection has resulted in reliable materials that are being widely used in industrial applications [11,12]. Epoxy resins are a frequently used coating material, particularly in protective primers, for severe corrosive environments and marine applications. Epoxy resins have excellent mechanical and electrical properties and are commonly used with carbon or glass fibers. They also have good electrical insulation properties in a wide range of applications [13].

Initially, epoxy resins were used to coat surfaces. Since 1980, the worldwide capacity of epoxy resins has been 600,000 tons per year, but at this time, the companies use only 50–60% of them. Considering that 10 million tons of thermoreactive resins are consumed annually in the world, epoxy resins occupy about 3%. Western Europe and the USA produce about 40% of the market need, while Japan accounts for about 10%. This situation has not changed much since then, so in the year 2000, the market for epoxy resins reached around 750,000 tons per year. Epoxy resins are considered the best synthetic resins due to their high modulus of elasticity and high mechanical strength. They are used in different fields: surface engineering (as additive agents), in painting, for encapsulation of electronic components, for lamination etc. They are excellent insulating materials, exhibiting high dielectric strength, electric arc resistance, good thermal stability (over 260 °C), very low water absorption and equally high tolerance to corrosion in various conditions [14,15,16,17,18,19,20].

In the present work, we report the influence of the current density and mean diameter size of epoxy resin particles used for electrodeposition on the morphology, topography and corrosion resistance of epoxy resin/zinc composite coatings obtained in a zinc sulphate electrolyte bath. In terms of surface observations, morphology was studied via Scanning Electron Microscopy (SEM), and topography and roughness were analyzed with Atomic Force Microscopy (AFM). The results show that hybrid coatings surfaces are smooth, uniform, compact and do not present irregularities. Corrosion resistance in 0.5 M sodium chloride was estimated using potentiodynamic polarization method and all hybrid coatings tested have very good corrosion resistance. The microbiological corrosion in the presence of SRB was evaluated using Epifluorescence Microscopy (EFM) and AFM The results of values of average roughness and maximum peak to valley height of the surfaces after attachment of bacteria and biofilm formation are higher compared with values before the attachment of bacteria.

Global demand for zinc has increased continuously such that current market demand has outstripped global production capability. Epoxy resins have been used for anticorrosive properties in paints and coatings for decades, but when embedded in a zinc matrix they have been shown to significantly decrease the corrosion rate of the naked metal. Thus, the methods proposed here could be readily applied at an industrial scale to sustainably increase the service lifetime of electrogalvanized zinc. The starting materials are not expensive, are non-toxic, and would have a low environmental impact if the epoxy employed is bisphenol A (BPA) free. Moreover, clean-up and recycling of the erased sulphate electrolyte bath is expected to be facile, as is wastewater treatment, because no surfactants are employed in the process.

## 2. Materials and Methods

### 2.1. Preparation of Epoxy Resin/Zinc Hybrid Coatings

Carbon steel sheets DC04 (Liberty Steel, Galaţi, Romania) were used as substrates. They were polished with abrasive papers, from 80 °C to 600 °C, degreased with an alkaline solution and washed with distilled water. The sample used as substrate had the following composition in percent weight: C = 0.04, Mn = 0.20, Si = 0.02, P = 0.018, S = 0.012, Al = 0.060 and Fe up to 100. For electrodeposition, a sulphate electrolyte was used, with the following composition: 1.078 mol/L ZnSO_4_·7H_2_O; 0.232 mol/L Na_2_SO_4_·10H_2_O; 0.045 mol/L Al_2_(SO_4_)_3_·18H_2_O. Sodium sulphate was used to increase the conductivity of the solution and aluminium sulphate was used to stabilize the electrolyte acidity. The electrolyte solution registered a pH of 3.8. Deposition was carried out at current densities of 40 mA/cm^2^ and 50 mA/cm^2^, electrodeposition time of 30 min, 10 g/L epoxy resin particles in zinc sulphate electrolyte solution with mean diameter size of particles 0.1–5 and 6–10 μm, stirring rate of 800 rpm at room temperature. Surfactants or additives were not used in the zinc sulphate electrolyte because they could react with resin particles. The epoxy resin (S.C. Azur S.A, Timişoara, Romania) used as the disperse phase has molecular weight 900–1200 g/mol, density 1.18–1.25 g/cm^3^, epoxy index 0.185–0.220 equivalents in 100 g resin 100%, melting point 64–76 °C and volatile substances content of maximum 1%. The obtained samples were submitted to experimental determinations and the symbols of tested samples are presented in Table 1.

### 2.2. Characterization of the Hybrid Coatings

#### 2.2.1. SEM and AFM Characterization

The morphology of deposits was examined by SEM using a microscope type Philips FEI; QUANTA 200 (FEI Company, now Thermo Fischer, Waltham, MA, USA). The thickness of the coating was evaluated via cross-section samples using optical microscopy and SEM. EDX analysis was used to evaluate inclusion of polymer particles in zinc matrix. The roughness and topography of the hybrid coatings were evaluated using JPK NanoWizard II Atomic Force Microscope (JPK BioAFM, Bruker Corporation, Berlin, Germany) performed by contact mode in air. A silicon cantilever CSC37 A with the following characteristics was used for AFM images: standard length of 250 μm; width of 35 μm; thickness of 2 μm; resonance frequency of 41 kHz as well as nominal force/ spring constant of 0.65 N/m. Each AFM image has a resolution of 512 × 512 pixels.

Roughness is a measure of surface topography and AFM is considered one of the most dominant technique for characterization of surface roughness because it is a non-vacuum method and the results are accurate [21,22,23,24]. The roughness parameters are estimated by analyzing the topography scans of the tested samples. The parameters for the surface profile usually involve average roughness (Ra), root mean square roughness (Rq), and peak to valley maximum height (Rt). The amplitude parameters of a sample are defined by characteristics that provide information on statistical average values, shape of histogram heights and other specific properties. The average roughness is the mean height as calculated over the entire length/ area analyzed. Generally, the average roughness has been used to characterize the roughness of machined surfaces. It is effective in the identification of general variability of total heights for the profile and thus for controlling an existing manufacturing system. Maximum peak to valley height roughness represents the vertical distance among both the maximum and minimum locations of the analyzed roughness of a surface. Root mean square (RMS) roughness is the square root of the surface height distribution and is regarded more important than the average roughness, especially in cases of wide ranging variations from mean line/plane [25,26].

#### 2.2.2. Corrosion Tests by Electrochemical Methods

Electrochemical measurements used to evaluate the corrosion resistance of coatings were made using a Voltamaster 4 (PGP 201) (Radiometer Analytical SAS, Villeurbanne, France). In order to evaluate the stability of the system in 0.5 M sodium chloride, all tests started with monitoring of corrosion potential (open circuit potential). Potentiodynamic polarization measurements were performed in a three-electrode open cell with epoxy resin/Zn hybrid coatings as working electrode (W.E.), a platinum plate as counter electrode (C.E.) and a saturated calomel electrode (SCE) as reference electrode (SCE = +241 mV/NHE). Initial potential (I.P.) was −1.7 V (Hg/Hg_2_Cl_2_), final potential (F.P.) was −0.6 V (Hg/Hg_2_Cl_2_) and a scan rate of 0.5 mV/s. The potentiodynamic polarization curves have been conducted in electrolyte solution after 30 min of immersion. The Tafel parameters were defined by extrapolating the Tafel anode as well as cathode curves of the specific samples. The reproducibility of the realized investigations is an average of three samples per specimen type.

#### 2.2.3. Microbiological Corrosion Tests

In order to evaluate corrosion resistance of the hybrid coatings under microbiology induced corrosion, cells suspensions with Sulphate Reducing Bacteria (*Desulfovibrio Vulgaris*) with a pH = 6.2 and a concentration of cells of about 10^9^ cells/mL were added on the tested samples. Attachment of cells was made in the following steps:

Step 1: putting a drop from preparate solution cells on the surface of coatings;
Step 2: waiting to dry (15–20 min);
Step 3: it was polymerized for 24 h with glutaraldehyde 2.5%;
Step 4: the biofilm formation of SRB on hybrid coatings surfaces was examined using epifluorescence microscopy. Sessile bacteria on coupons were stained with 0.01% DAPI (4′,6–diamidino–2–phenylindol) for 10 min and visualized by epifluorescence microscopy. In epifluorescence microscopy, excitation filter wheels control the illumination spectrum before it enters the objective, whereas emission filter wheels are mounted on the camera port and select a unique emission wavelength.

Biofilms and attached cells on hybrid coatings tested were investigated using EFM coupled with AFM. The EFM was used to evaluate the attachment of the SRB on the surfaces and AFM was used to evaluate the surface roughness after bacterial attachment.

## 3. Results and Discussions

### 3.1. Morphology and Topography of the Hybrid Coatings

**Figure 1:**
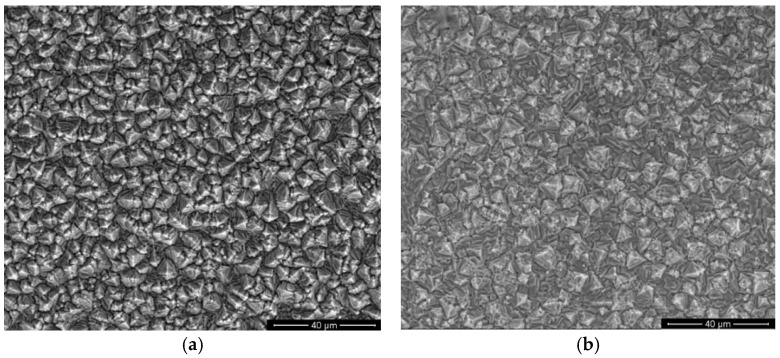

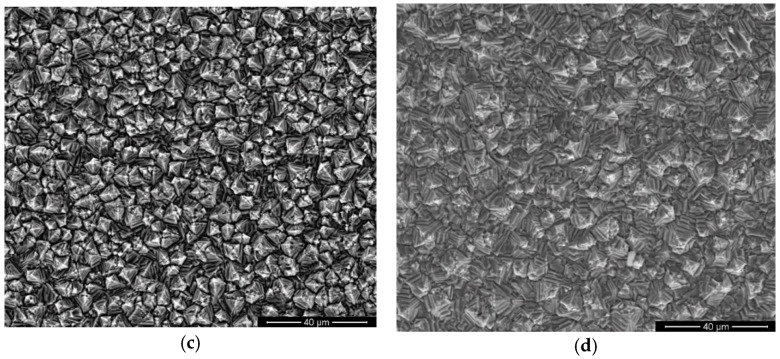
SEM Scanning electron microscopy of surface morphology of epoxy resin/zinc hybrid coatings electrodeposited at different parameters: (**a**) sample A; (**b**) sample B; (**c**) sample C; (**d**) sample D. Magnification: 2000×.

Figure 1a–d compare morphological aspects of epoxy resin/zinc hybrid coatings under the scanning electron microscope.

As shown through the micrographs, there is a visible and distinctive grain rearrangement on the surface of hybrid coatings in presence of the polymer particles. It is probable that the polymeric particles initially attach to the growing macrosteps of the planes and subsequently drift along and are engulfed between stacked layers of the metal. Nucleation sites increment is observed, meaning that the polymer particles possess an inhibition effect in crystal growth of Zn and catalytic activity. Accordingly, the typical stacking sequence of hexagonal platelets of pure zinc coatings converts to a micro-heterogeneous morphology with a smaller metal grain size. The same layer morphology was observed from electrodeposition of zinc with PMMA particles [10].

**Figure 2:**
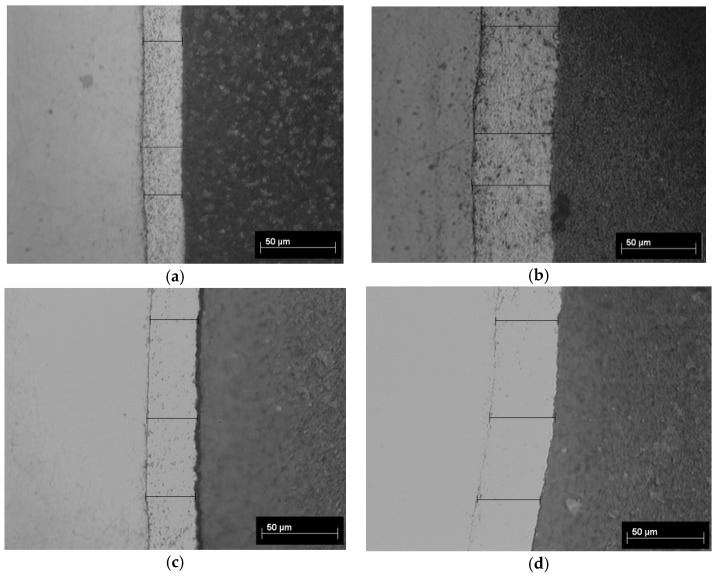
Cross-section images obtained with optical microscopy showing the thickness of epoxy resin/zinc hybrid coatings: (**a**) sample A; (**b**) sample B; (**c**) sample C; (**d**) sample D. Magnification: 500×.

**Figure 3:**
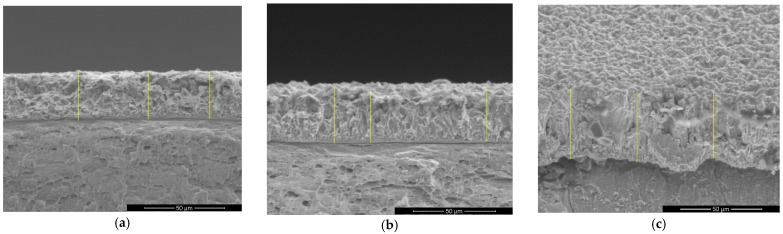
Cross-section images obtained with SEM showing the thickness and morphology of epoxy resin/zinc hybrid coatings: (**a**) sample A; (**b**) sample C; (**c**) sample D. Magnification: 2000×.

Cross-section images obtained with an optical microscope showing the coating thicknesses are presented in Figure 2a–d and cross-section images obtained with SEM are presented in Figure 3a–c.

From the values of the layer thicknesses presented in Table 2 it could be observed that with increasing of current density from 40 mA/cm^2^ to 50 mA/cm^2^ the thickness of the hybrid layers increases only for particles with mean diameter size of 0.1–5.0 μm. For polymer particles with 6–10 μm mean diameter size, the thickness of the hybrid layers obtained by electrodeposition does not increase with current density, exhibiting an inverse trend with an observable decrease in thickness. Probably, the polymer particles in this case do not act as a catalyst for zinc crystals growth and the surfaces are not compact and uniform like in the other studied cases.

To evaluate the presence of epoxy resin particles in the zinc matrix, EDX analysis was carried out. The epoxy resin contains hydrogen, oxygen and carbon. The presence of hydrogen cannot be determined because it has a very small atomic weight. The average elemental (carbon, oxygen and zinc) composition of the coatings was used to evaluate the inclusion of polymer in zinc matrix. The average content of unit molecular weight of polymer from hybrid coatings was calculated using the average content of carbon and described in Table 3.

**Figure 4:**
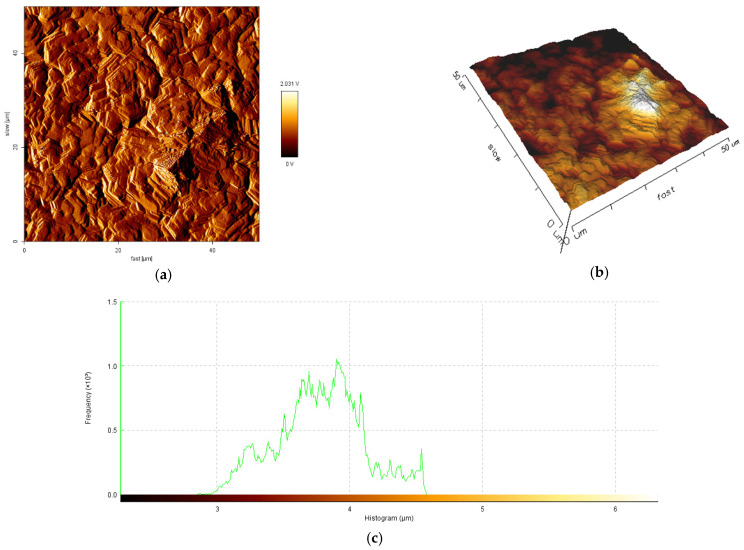
50 × 50 μm^2^ Atomic Force Microscopy (AFM) images of sample A: (**a**) 2D image; (**b**) 3D image; (**c**) histogram of the scanned surfaces.

**Figure 5:**
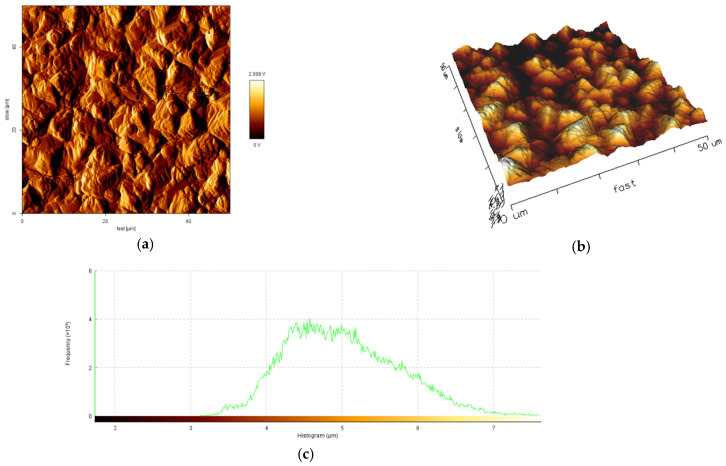
50 × 50 μm^2^ AFM images of sample B: (**a**) 2D image; (**b**) 3D image; (**c**) histogram of the scanned surface.

**Figure 6:**
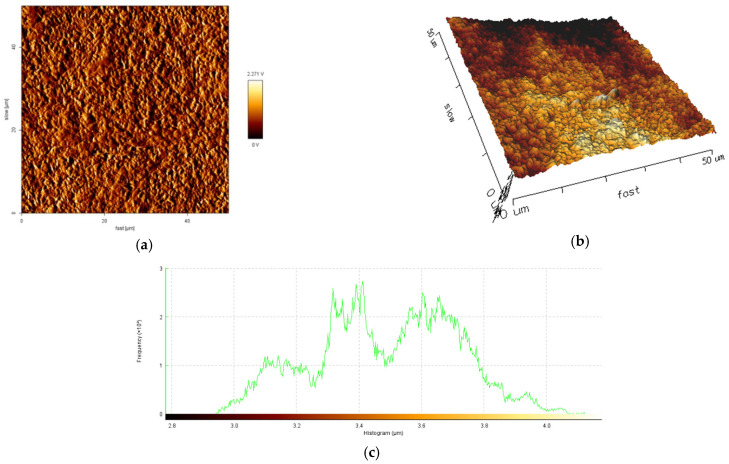
50 × 50 μm^2^ AFM images of sample C: (**a**) 2D image; (**b**) 3D image; (**c**) histogram of the scanned surfaces.

**Figure 7:**
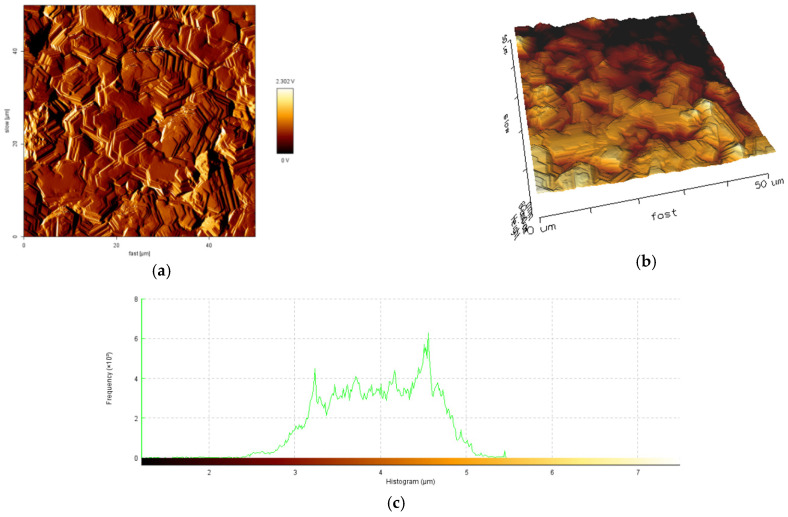
50 × 50 μm^2^ AFM images of sample D: (**a**) 2D image; (**b**) 3D image; (**c**) histogram of the scanned surfaces.

The influence of current density and the mean diameter size of polymer particles used for electrodeposition on topography and roughness of the polymer/zinc surfaces are presented in Figure 4, Figure 5, Figure 6 and Figure 7.

By increasing the current density for obtained hybrid coatings with mean diameter size of particles 0.1–5.0 μm it could be observed that layers become uniform, with smaller grains, randomly orientated. By increasing the mean diameter size of polymer particles and maintaining current density at 40 mA/cm^2^ it could be observed that the crystal grains are bigger, which in general leads to a surface roughness increase. The reinforcing particles in zinc matrix are not concentrated at a particular region, all the surfaces are smooth, compact, do not present irregularities and the hybrid composites layers are uniform.

From the histograms of the scanned surfaces, different roughness parameters (R_a_, R_q_ and R_t_) were evaluated and their values were presented in Table 4.

The obtained R_a_ values are in a range of 190.4 nm to 598.1 nm, with higher values for samples obtained with particles of 6–10 μm mean diameter size. This aspect shows that the hybrid coating surfaces obtained with particles of higher mean diameter size are rougher than the hybrid coating surfaces obtained with particles of 0.1–5.0 μm mean diameter size. The AFM analysis suggested that using polymer particles with particles of smaller mean diameter size could decrease the roughness of coatings.

The R_q_ of tested samples varies from 226.7 nm to 735.7 nm, indicating that the degree of surface roughness variation in epoxy resin/zinc samples with 6–10 μm mean diameter size of polymer particles are higher than that in epoxy resin/zinc samples with polymer particles of 0.1–5.0 μm mean diameter size. From the values of R_t_ it could be concluded that coatings with mean diameter size of polymer particles 6–10 μm could be fissured because they have a higher value of R_t_ from all tested types and if the total height of the profile is higher, the surfaces are capable of generating crack propagation.

### 3.2. Electrochemical Corrosion Measurements

**Figure 8:**
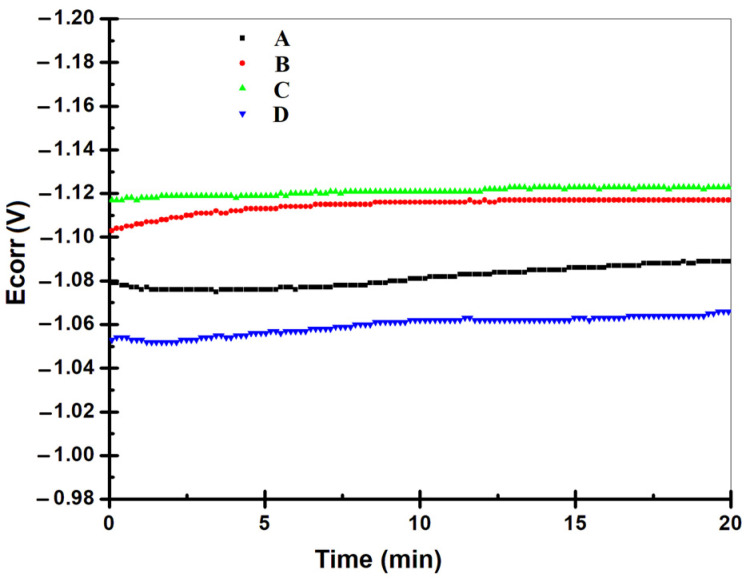
Open circuit potential measurements of the tested samples.

The results obtained for the changes of the open-circuit potential values of all tested samples are presented in Figure 8. The results obtained indicate a corrosion potential range from −1.05 V to −1.12 V, variation of the corrosion potential in time being very slow. A thorough examination reveals that the curves of corrosion potential, known as open circuit potential, vs time appears nearly straight, indicating that steady state potential is created. Furthermore, it can also be seen that for hybrid coatings obtained at 50 mA/cm^2^ and epoxy resin particles with mean diameter size of 6–10 μm, open circuit potential (OCP) versus time curves shifted towards the more positive (anodic) direction.

**Figure 9:**
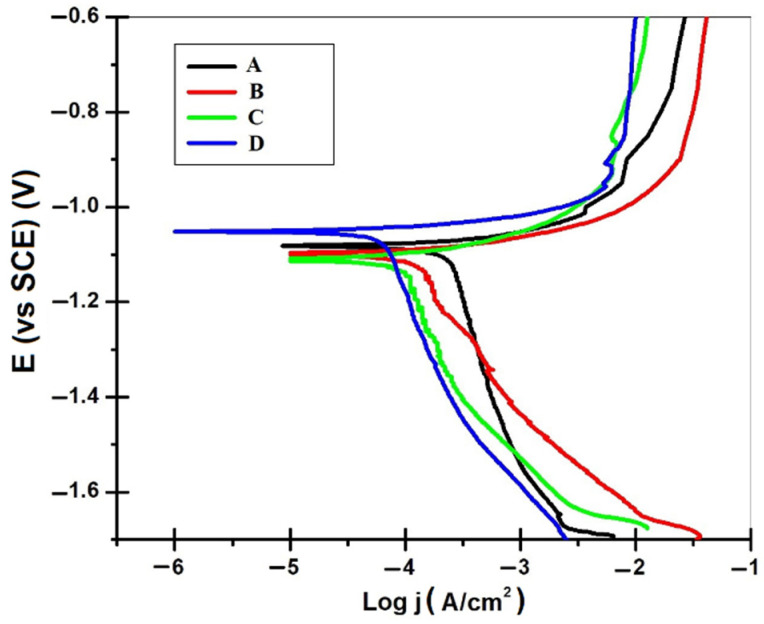
Tafel plots for hybrid coatings in 0.5M sodium chloride solution.

The polarization measurements were carried out in the potential range from −1.7 to −0.6 V using a corrosive medium of 0.5 M sodium chloride solution. The Tafel plots of tested samples are given in Figure 9. The corrosion parameters such as corrosion potential (*E*_corr_), corrosion current density (*j*_corr_), corrosion rate (*CR*) and anodic/cathodic Tafel slopes (*β*_a_ and *β*_c_) derived from the slope of the curve according to the Stern-Geary equation [27,28] are tabulated in Table 5.

Comparing the tested samples, it can be clearly found that all polarization curves had similar shapes, implying similarities in polarization behavior and corrosion mechanism. The exposed specimens had undergone corrosion to different extents, depending on the different parameters used for obtaining these hybrid coatings by electrodeposition. Nonetheless, for exposed specimens, both the anodic and cathodic processes were apparently inhibited, meaning that the corrosion products formed on epoxy resin/zinc hybrid coatings surface could offer protection against the aggressive medium.

From the Tafel parameters it could be observed that the smaller corrosion rate was evaluated for epoxy resin hybrid coatings obtained at 40 mA/cm^2^, with mean diameter size of particles 0.1–5.0 μm and with a value of 7.65 μm/year. For the other tested samples, the corrosion rate was in range of 20.71 μm/year to 27.48 μm/year.

Some reports indicate that the corrosion rate in 5% NaCl solution of hybrid coating Zn–PANI–SiO_2_ covered with Pluronic F127 has a value of 150 μm/year, hybrid coating Zn–PANI–SiO_2_ covered with Pluronic F127 and benzalacetone in ethanol used as additive for electrodeposition has a corrosion rate of 165 μm/year and ordinary zinc electrodeposits in the same condition has a corrosion rate of 240 μm/year [9]. For other hybrid coatings with zinc matrix and polymers used as disperse phase, it was reported for composite Zn(PMMA), particle-free Zn(00.2) and commercial galvanized steel coatings in aerated NaCl 0.6 M solution, a corrosion rate with values of 102 μm/year, 122 μm/year and 212 μm/year [10].

Comparing the values of corrosion rate obtained for epoxy resin/zinc hybrid coatings with the values of corrosion rate for zinc and other coatings obtained from zinc and polymer as disperse phase reported in the literature, it could be concluded that epoxy resin/zinc hybrid composite coatings have a very good corrosion resistance in an aggressive environment. More than that, these coatings could be used for industrial purposes to decrease the losses of zinc generated from corrosion, because the method of obtaining is easier, with low equipment cost and starting materials.

### 3.3. Microbiological Corrosion Tests

**Figure 10:**
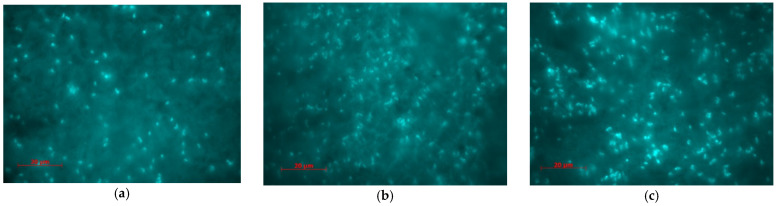
Epifluorescence Microscopy (EFM) image of the Sulphate reducing bacteria (SRB) attachment and extracellular polymeric substances (EPS) formed on tested samples surfaces: (**a**) sample A, (**b**) sample B, (**c**) sample C.

To describe the bacterial attachment on the coating surfaces, the fluorescence observations were performed on samples. Attachments of bacteria on different surfaces create a biofilm formed by microbial aggregates and extracellular polymeric substances (EPS) including exopolysaccharides, proteins and DNA. The production of EPS is important for the survival and development of biofilm that will influence the corrosion of the surface on which the bacteria are attached. The blue light points/areas in the fluorescence images in Figure 10a–c represent the bacteria bodies/colonies attached to the surfaces.

From the epifluorescence images it could be observed that a lower quantity of bacteria was attached on epoxy resin/Zn hybrid coating obtained at 40 mA/cm^2^, with particles of 0.1–5.0 μm mean diameter size. For the other tested samples, the attachment of SRB has the same aspects. The values of roughness calculated from histograms after attachment of SRB on the hybrid coating surfaces indicate values of R_a_ = 310.1 nm, R_t_ = 3.303 μm for sample A and R_a_ = 1.132 μm, R_t_ = 6.296 μm for sample C, that indicate an increase in values of average roughness and maximum peak to valley height of the surfaces after attachment of bacteria and biofilm formation.

## 4. Conclusions

In this research, the effect of current density and mean diameter size of epoxy resin particles used for electrodeposition in zinc sulphate electrolyte, on morphology, topography, roughness and anticorrosion properties of hybrid epoxy resin/zinc coatings on steel is evaluated. The key results are outlined as follows:

Epoxy resin particles used for obtaining hybrid coatings do not affect the electrodeposition process; they only modify the morphology and topography of the layer’s surfaces. The hybrid coating surfaces are smooth, uniform, compact and do not present irregularities. Polymer particles provide an influence in restricting the growth of zinc crystals as well as a catalytic role in nucleation sites increase.

The values of roughness parameters for obtained samples show that the hybrid coating surfaces with the higher mean diameter size of polymer being rougher than those of the hybrid coating surfaces with smaller mean diameter size. The atomic force microscopy analysis suggested that using epoxy resin particles with smaller mean diameter size could decrease the roughness of coatings.

From potentiodynamic polarization curves it could be concluded that all of them had similar shapes, implying similarities in polarization behavior and corrosion mechanism. All hybrid coatings tested in 0.5 M sodium chloride have very good corrosion resistances. More than that, these coatings could be used for industrial purposes to decrease the losses of zinc generated from corrosion, the method obtained is easier, with low costs for equipment and starting materials.

Epifluorescence images demonstrate that hybrid coating surfaces are slightly attacked by sulphate reducing bacteria, and these coatings have a good microbiological corrosion behavior. Values of average roughness and maximum peak to valley height of the surfaces after attachment of bacteria and biofilm formation are higher compared with values before the attachment of bacteria.

## Figures and Tables

**Table 1:** Symbols of samples used for testing.

Symbols	Electrodeposition Parameters
A	40 mA/cm^2^, mean diameter size of polymer particles 0.1–5.0 μm
B	40 mA/cm^2^, mean diameter size of polymer particles 6–10 μm
C	50 mA/cm^2^, mean diameter size of polymer particles 0.1–5.0 μm
D	50 mA/cm^2^, mean diameter size of polymer particles 6–10 μm

**Table 2:** The thickness of epoxy resin/zinc hybrid coatings.

Type of Samples	Thickness of the Hybrid Coatings Layers (μm)
A	30.76
B	50.11
C	32.47
D	41.23

**Table 3:** Composition of hybrid coatings obtained from EDX analysis.

Type of Samples	Average Composition of Carbon	Average Composition of Oxygen	Average Composition of Zinc	Mean Value of wt%
A	16.75	5.60	77.65	22.02
B	14.26	3.72	82.02	18.92
C	7.08	3.37	89.55	9.31
D	10.24	3.23	86.53	13.46

**Table 4:** Roughness parameters for tested samples.

Type of Samples	Roughness Parameters		
	R_a_ (nm)	R_q_ (nm)	R_t_ (μm)
A	259.3	326.1	1.714
B	598.1	735.7	4.469
C	190.4	226.7	1.178
D	490.5	581.3	4.259

**Table 5:** Tafel parameters of hybrid coatings determined from potentiodynamic polarization curves obtained in 0.5 M NaCl after 30 min of immersion.

Type of Samples	E_corr_ (vs SCE) (V)	β_a_ (V/decade)	|β_c_| (V/decade)	j_corr_·10^−6^ (A/cm^2^)	R_p_ (Ω·cm^2^)	v_corr_ (μm/year)
A	−1.08 ± 0.12	0.018 ± 0.003	0.246 ± 0.044	16.23 ± 1.17	462.71	7.65
B	−1.10 ± 0.17	0.030 ± 0.002	0.103 ± 0.023	43.91 ± 2.75	235.39	20.71
C	−1.11 ± 0.21	0.032 ± 0.003	0.086 ± 0.017	58.26 ± 3.18	176.62	27.48
D	−1.05 ± 0.19	0.027 ± 0.003	0.092 ± 0.021	46.14 ± 2.95	201.25	21.76

## Data Availability

The data presented in this study are openly available.
